# Anthropogenic Drivers of Small-Island Effects in Urban Remnant Woody Plants

**DOI:** 10.3390/plants13243522

**Published:** 2024-12-17

**Authors:** Di Kong, Kai Wang, Lin Dong, Jinming Yang, Zhiwen Gao, Hong Liang

**Affiliations:** 1 College of Landscape Architecture and Forestry, Qingdao Agricultural University, Qingdao 266109, China; 20212110009@stu.qau.edu.cn (D.K.); dlwangkai@163.com (K.W.); jinming0221@qau.edu.cn (J.Y.); 2 School of Landscape Architecture, Nanjing Forestry University, Nanjing 210037, China; dlinoo@163.com; 3 Zhejiang Tiantong National Forest Ecosystem Observation and Research Station, School of Ecological and Environmental Sciences, East China Normal University, Shanghai 200241, China; 4 Institute of Eco-Chongming, Shanghai 200062, China

**Keywords:** urban biodiversity, remnant vegetation, habitat fragmentation, small island effect, species–area relationship

## Abstract

The positive relationship between species richness and area is a fundamental principle in ecology. However, this pattern deviates on small islands, where species richness either changes independently of area or increases at a slower rate—a phenomenon known as the Small-Island Effect (SIE). While the SIE has been well documented in natural ecosystem, its presence in highly fragmented and disturbed urban ecosystem remains unexplored, posing challenges for urban vegetation conservation. Urban remnant vegetation, isolated by surrounding infrastructures, preserves intact zonal vegetation characteristics, serves as a benchmark for restoring near-natural habitats and offers ideal conditions to test the existence of the SIE in urban area landscapes. In this study, we surveyed 17 remnant vegetation patches in Qingdao City, China. A total of 331 plants attributed to 255 genera in 81 families have been recorded. Firstly, by using six species–area relationship regression models testing the SIE for remnant vegetation with different plant life forms, we found the SIE in only woody plants, with the land surface area threshold ranging from 6.38 ha (tree) to 11.91 ha (shrub). Our finding revealed that the drivers of the SIE in shrubs were landscape shape index, perimeter–area ratio, and the proportion of sealed surfaces within the patch. For trees, the SIE was influenced by the distance to the source of species, *GDP*, night light intensity, and perimeter–area ratio. This finding justifies that conservation in urban planning, construction, and development should focus not only on protecting large areas but also on maintaining and promoting diverse habitats within these areas. At the same time, reducing anthropogenic disturbance and enhancing the connectivity of green spaces are important for the persistence of metacommunities and can contribute to the local species pool, thus potentially improving the ecological resilience of urban environments.

## 1. Introduction

As urbanization accelerates, natural habitats within and surrounding cities are increasingly being replaced by artificial green spaces or becoming fragmented, leading to a significantly decline in biodiversity [1]. A habitat island refers to a discontinuous island-like habitat that restricts the exchange and gene flow of individuals of the same species. The size, shape, and degree of isolation of the habitat island have an important influence on the island biome, giving it characteristics different from those of the “mainland” biome [2]. In some cities with unique geomorphological features, some remnants of natural vegetation are still preserved, becoming habitat islands in the city, which are distributed in the form of isolated islands [3]. Most of these remnant vegetation patches are natural hills that are difficult to develop and utilize, allowing for the preservation of remnant vegetation. These remnant vegetation patches are adapted to local climatic conditions and function as a vital seed bank for ecological succession, as well as important resources for the restoration of near-natural habitats in urban design [4]. Additionally, these urban remnant vegetation patches provide essential habitat for urban wildlife and offer various ecosystem services [5,6,7], whereas the surrounding urban areas are mostly flat and dominated by reinforced concrete, resulting in a monotonous habitat. However, extensive habitat fragmentation has severely disrupted the natural vegetation pattern, leaving intact patches of remnant vegetation scarce and widely scattered across the city [8,9]. The fragmentation of these habitats not only disrupts ecological balance but also diminishes the resilience of urban ecosystems to environmental changes, underscoring the urgent need for conservation efforts in recent years [10,11,12,13]. Since species–area relationships may vary among plant life forms due to their various responses to environmental factors, such as woody plants being potentially more sensitive to habitat properties and urbanization intensity [14], understanding these factors in relation to the different life forms of vegetation is crucial. Such knowledge can inform the development of effective strategies to preserve biodiversity in urban environments and mitigate the negative impacts of urbanization.

Island biogeography theory (IBT) is a cornerstone of conservation biology, offering valuable insights into the effects of habitat fragmentation [15]. The concept of IBT has been proven invaluable for understanding patterns of biodiversity in urban ecosystems. Urban green spaces, often isolated by impervious surfaces and built structures, can be conceptualized as islands within a sea of urban matrices [16,17]. The characteristics of this matrix, such as its permeability and the presence of corridors, can significantly influence the movement of organisms between these urban “islands” and, consequently, their biodiversity [18]. As one of the focal points of research on island biogeography, the Small Island Effect (SIE) describes a phenomenon in which, when below a certain threshold area, species richness is no longer directly proportional to island area and may even decrease [19]. The SIE is particularly relevant for small-population conservation, as small populations are more susceptible to stochastic events, and the SIE highlights the vulnerability of island ecosystems to species extinction [20]. In urban and landscape ecology, SIEs can provide a theoretical foundation for understanding species distribution patterns in fragmented landscapes, aiding in the assessment of habitat quality at various scales [21]. Area thresholds based on SIE emergence allow for more scientific planning of green spaces. With the intensification of climate change and human activities, grasping the patterns of SIE existence may have a positive effect on the conservation of urban biodiversity and the maintenance of ecosystem stability [22]. Therefore, a deep understanding of the SIE is crucial for predicting future trends in biodiversity [23].

Several hypotheses have been proposed to explain the SIE, such as (1) habitat diversity: smaller islands may experience fewer species deficits due to higher habitat diversity per unit area [24,25]; (2) extinction: catastrophic events on small islands can lead to total extinctions, preventing species richness from reaching equilibrium levels [26]; (3) nutrient replenishment: exogenous resources can increase island productivity, affecting species richness. Smaller islands may benefit more from nutrient replenishment per unit area [27]; (4) disturbance: small islands may be in different stages of disturbance recovery, supporting a wider range of species [28]. While these hypotheses have been explored in various contexts, the existence of the SIE in urban remnant vegetation and its underlying factors remain largely understudied.

Qingdao is a hilly coastal city with rich flora; it has undergone rapid urbanization over the past few decades, yet remnants of natural mountainous areas remain within the urban landscape. Its unique location and swift urbanization offer an ideal setting to study the SIE, particularly in remnant natural vegetation. Therefore, we selected 17 remnant vegetation patches in the urban area of Qingdao and surveyed the species diversity pattern of remnant vegetation, and by using six species–area relationship regression models, we tested whether the SIE exists for remnant vegetation with different life forms (i.e., annual herb, perennial herb, shrub, and tree). For those life forms where an SIE was detected, we also determined the threshold area values. Finally, a stepwise and generalized linear regression model was used to analyze the driving factors behind the generation of SIE with different life forms. Our objectives were the following: (1) to detect the presence of the SIE across different life forms in remnant vegetation patches; (2) if present, to identify the drivers of the SIE for different life forms and explore the differences among them. These findings are expected to offer valuable insights for optimizing urban green space planning and preserving biodiversity in rapidly urbanizing landscapes.

## 2. Results

### 2.1. Species Composition

**Figure 1:**
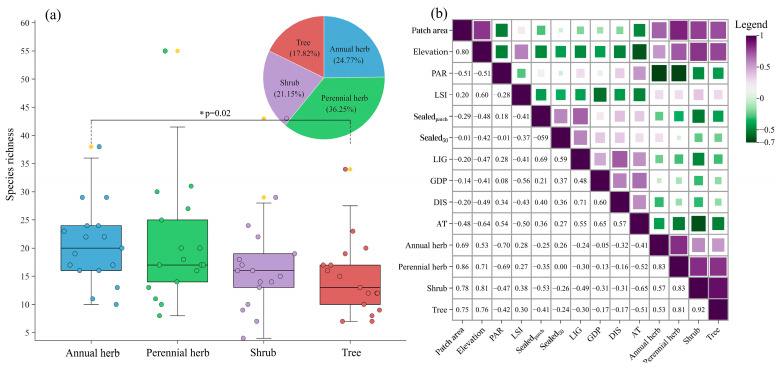
(**a**) Box plot of species richness in 17 remnant vegetation patches. Yellow point—outlier point. Except for yellow, the different coloured dots represent the species richness values of different life forms in each of the 17 remnant vegetation patches. (**b**) Correlation between species compositions and environmental factors in 17 remnant vegetation patches. *PAR*—perimeter–area ratio; *LSI*—landscape shape index; *Sealed_patch_*—impervious surface inside the patch; *Sealed*_50_—the proportion of sealed surface around the patch within a radius of 50 m; *Light*—night-time light index; *GDP*—Gross Domestic Product; *DIS*—the shortest straight-line distance of each remnant vegetation patch from the source of species—Mt. Laoshan; *AT*—anthropogenic disturbance; remnant vegetation of different life forms: annual herb, perennial herb, shrub, and tree.

Across the 17 remnant vegetation patches in Qingdao city, we recorded 331 species of 225 genera and 81 families, including 24.77% annual herbaceous species, 36.25% perennial herbs, 21.15% shrub species, and 17.82% tree species (Figure 1). These species included 248 native plants and 83 non-native plants; in addition, we identified 38 invasive plants. Across all patches, the proportion of herbaceous species (mean = 56.99%, range from 35.71% to 70.18%) exceeded that of woody plants (mean = 43.01%, range from 29.83% to 64.29%). Within herbaceous plants, the proportion of annual herbs among patches (mean = 51.37%, range from 44.44% to 66.67%) exceeded that of perennial herbs (mean = 48.63%, range from 33.33% to 59.14%). Pearson correlation analysis showed that there was a significant correlation between annuals and tree (*p* = 0.02), while there was no significant correlation between any of the other taxa (Figure 1). The highest and lowest richness of species recorded in a single patch were 170 and 33, respectively, whereas the mean species richness was 73. Of all species, the top three frequency families were Asteraceae (50 species), Gramineae (28 species), and Rosaceae (24 species), containing about 38.4% of the total remnant vegetation species. The top two frequency genera were *Artemisia* (nine species) and *Erigeron* (five species). In these 17 patches, 13 species were observed to occur with a frequency of 88%. Such as, *Trigonotis peduncularis*, *Oxalis corniculata*, and *Robinia pseudoacacia*. The number of species that occurred in only 1 patch was 142.

### 2.2. Detection of the Small Island Effect

**Figure 2:**
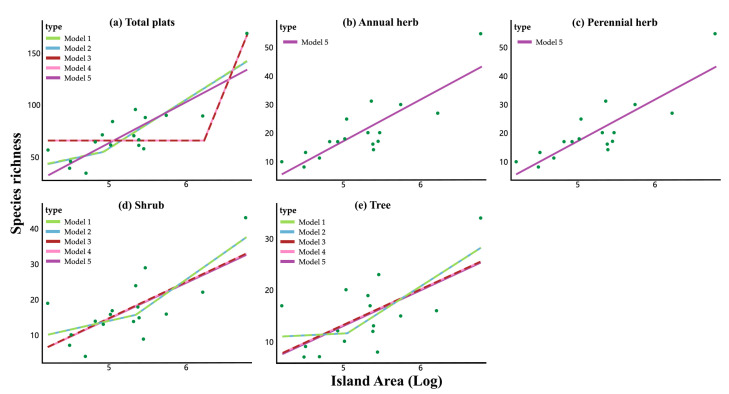
Best-fitting model results for remnant vegetation patches with different life forms. Model as in Table 3. For annual (**b**) and perennial (**c**) herb, Model 5 is the best model; for total plants (**a**), shrubs (**d**), and trees (**e**), Model 1 to Model 5 have similar explanatory power.

The fitting results indicate that annual and perennial herbs do not exhibit the small island effect (SIE), as evidenced by Model 5 being the only optimal model (∆AICc > 2 compared to suboptimal models), suggesting stable species richness across areas and no significant SIE. In contrast, for trees and shrubs, Models 1 to 5 show similar explanatory power (∆AICc < 2), indicating the presence of the SIE. The area thresholds for the presence of the SIE are 6.38 ha for trees and 11.91 ha for shrubs. Additionally, for total plant species, Models 1 to 5 also have similar explanatory power, with an area threshold of 86.44 ha (Table 1, Figure 2).

### 2.3. Model Analysis

In our analysis of the drivers behind the SIE across different plant life forms, we found that the drivers of the SIE vary among plant life forms. For total plants, sealed surfaces around the patches within a radius of 50 m (*Sealed*_50_) showed a consistent downward trend, which suggests that smaller patches are more vulnerable to the negative effects of surrounding impervious surfaces. In contrast, *Elevation*, the perimeter–area ratio (*PAR*), and sealed surface within patches (*Sealed_patch_*) showed a continuous upward trend, indicating that species diversity increases with patch size as these variables increase. For distance to the source of species (*DIS*), *GDP*, and the landscape shape index (*LSI*), initially a sharp decline was observed followed by a relatively slower increase, meaning these factors exert a strong influence on species diversity in smaller patches but stabilize as the area grows. Notably, anthropogenic disturbance (*AT*) displayed a significant downward trend followed by a marked upward shift, indicating a more dynamic relationship with the vegetation patches. Meanwhile, night light intensity (*Light*) exhibited a gradual upward trend followed by a slow decline, reflecting a more moderate but steady impact across patches of varying sizes.

For shrubs, apart from *LSI* and *Sealed_patch_* which consistently declined, all other variables exhibited a trend of initial decline followed by an increase, though the patterns varied across different factors. Specifically, *DIS*, *GDP*, *Sealed*_50_, and *Light* followed a trend of slowly decreasing and then a gradual increase. *Elevation* and *AT* exhibited a slow decline followed by a sharp increase, while *PAR* displayed a sharp decline followed by a slow rise. These patterns indicate that these variables initially exert a negative impact on shrub diversity especially for smaller patches.

**Figure 3:**
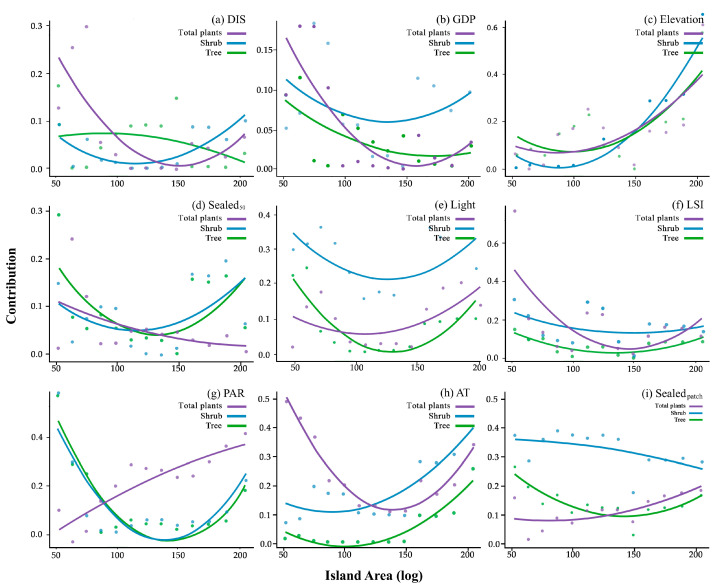
Trends in the contribution of factors. (**a**) *DIS*—the shortest straight-line distance of each remnant vegetation patch from the source of species—Mt. Laoshan; (**b**) *GDP*—Gross Domestic Product; (**d**) *Sealed*_50_—the proportion of sealed surface around the patch within a radius of 50 m; (**e**) *Light*—night-time light index; (**f**) *LSI*—landscape shape index; (**g**) *PAR*—perimeter area ratio; (**h**) *AT*—anthropogenic disturbance; (**i**) *Sealed_patch_*—impervious surface inside the patch.

In the case of trees, aside from *DIS* and *GDP* which showed a continuous decline, all other variables also exhibited an initial decline followed by an increase, though with varying patterns. *Light* and *PAR* experienced a sharp decrease followed by an increase. *Sealed*_50_, *LSI*, and *Sealed_patch_* showed a trend of decreasing slowly and then increasing, and *Elevation* and *AT* showed a trend of decreasing slowly and then increasing sharply, reflecting their variable but growing importance as patch size increase (Figure 3).

## 3. Materials and Methods

### 3.1. Study Area

The study area is located in Qingdao (119°30′–121°00′ E, 35°35′–37°09′ N), a major sub-provincial city in eastern Shandong province, China, with a total land area of 24,900 ha and about 10.37 million people in 2024. Qingdao has a temperate monsoon climate, and the urban area is directly regulated by the oceanic environment, thus displaying a number of distinctive oceanic climatic features: humid air, abundant rainfall, appropriate temperatures, and four distinctive seasons. Qingdao is one of the richest areas in plant species at the same latitude and was ranked as the “Forest city” in 2015, with a forest cover rate of 44.7% within built-up area [29]. The vegetation types in Qingdao can be divided into coniferous forests, deciduous broad-leaved forests, mixed coniferous broad-leaved forests, scrub, tussock, aquatic vegetation and cultivated vegetation. The vegetation of Mt. Laoshan, which can serve as a species source for Qingdao’s built-up area, belongs to the warm-temperate, deciduous, broad-leaved forest zone in China’s vegetation zoning. Qingdao is a hilly city; Mt. Laoshan extends to the main urban area, which has led to a special pattern of vegetation habitat fragmentation in the main urban area under the effect of rapid urbanization. Historically, there were a wide range of natural hills with remnant vegetation in Qingdao’s built-up area. With urban expansion, many of these hills have been converted into urban land, while some have remained throughout the city due to challenges in the developing process. However, in recent years, some of these natural remnant hills have also been gradually exploited through agricultural practices, horticultural activities, and the paving of impermeable surfaces. It creates problems for the conservation of the remnant vegetation.

**Figure 4:**
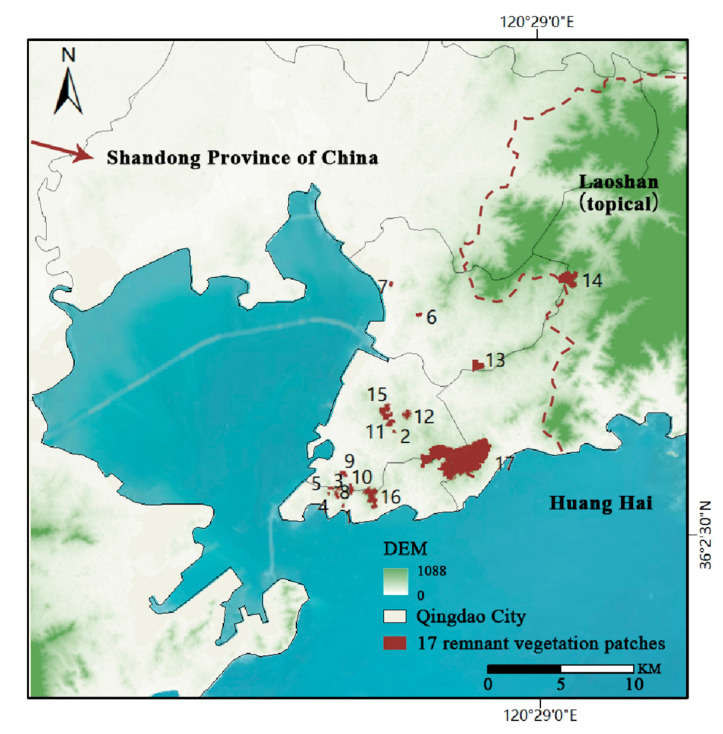
The distribution of remnant vegetation in Qingdao City, China. 1–17, numbering of remnant vegetation patches.

Our study is based on a randomized sampling; 17 remnant vegetation patches located in Qingdao were surveyed, with a mean area of 58.97 ha, ranging from 1.61 ha to 588.00 ha, and altitudes between 62 m and 384 m above sea level. These patches are isolated by surrounding urban buildings, scattered across the city, and face varying degrees of fragmentation. Situated on the edge of Qingdao, Mt. Laoshan serves as a key biodiversity reservoir for the region, acting as a “mainland” source of species for the isolated urban patches. Given its role as a species pool, we hypothesize that the distance and isolation from Mt. Laoshan significantly influence the species composition and richness within the remnant patches, providing a valuable opportunity to explore patterns in species diversity through the lens of island biogeography theory (Figure 4).

### 3.2. Field Surveys

We conducted plant species surveys in April-June and September-November of 2022 and 2023 in 17 remaining urban vegetation patches. Remnant vegetation refers to the plant populations that grow on remnant vegetation patches, which represents the local natural vegetation types and serves as a reference for future restoration efforts in the area. These plants are adapted to local climatic and soil environments, enabling them to support significant biodiversity and making them excellent subjects for SIE research.

During the plants survey, to ensure that as many species as possible were recorded in each patch, we established two sample lines across each remnant vegetation patch, with the total length of the sample lines being at least 1.5 times the perimeter of the surveyed patch. Each sample line of each patch was surveyed by at least two experienced researchers. Plant identification was conducted by a single researcher across all patches, reducing bias in identification. This identification process followed the standard in the *Flora of China* (https://www.iplant.cn/ (accessed on 10 April 2022)). Surveyed plants were further divided into annual herbs, perennial herbs, trees, and shrubs according to life form (classification is based on the *Vegetation of China*, 1980 [30]). We further classified the remnant vegetation into native, non-native, and invasive species based on the *Flora Laoshan* [31], *Exotic plants in China* (https://www.cvh.ac.cn/ (accessed on 10 April 2022)), and *The Checklist of Chinese Invasive Plants* [32].

### 3.3. Data Analysis

In this study, nine variables were selected for both the patch attributes and urbanization attributes to investigate the SIE of remnant plant patches in Qingdao. For patch attributes, *Elevation*, *PAR*, and *LSI* were included. For urbanization attributes, the variables included *DIS*, *Sealed_patch_*, *Sealed*_50_, *AT*, *Light*, and *GDP*. Four types of *AT* are typically observed and recorded in remnant patches, i.e., horticultural activities, laying of impervious surfaces, pruning and weeding, and agricultural activities [33]. The more of these categories present within the same patch, the higher the *AT* value (Table 2).

We compared the six continuous piecewise regression models to test for the existence of an SIE (Equations (1)–(6)); Table 3 [35]. The Akaike information criterion (AIC) was used in this study to evaluate the excellence of the model fit data. Two models are considered to have the same explanatory validity if the difference between their AICc values is less than 2 [20]. Continuous segmented regression models were selected for Models 1–4, and Models 5 and 6 were control models. If Models 1–4 are selected as the best models, it suggests the presence of an SIE [36]. Model 5 (S = C + Z_1_ × log A) indicates that species richness (S) is best explained by the combination of a constant (C) and the log-transformed area (A), with a coefficient (Z_1_) that reflects the strength of the relationship between the area and species richness. By using log-transformation, we address the non-linear nature of species–area relationships, allowing for a more accurate representation of the data. Where an island effect was identified, the average of the t-values (break point) obtained from the optimal segmented regression model was subsequently calculated as the area threshold for that data set.

The effects of each variable on changes in species richness were assessed separately using multiple linear regression and variance decomposition analyses and through an iterative approach. Area and each environmental variable were first log-transformed to enhance normality and variance alignment of the data. The five smallest islands were used to construct the first dataset, and a binary linear regression model was constructed with species richness as the dependent variable and area separately from each environmental variable. Subsequently, multiple linear regression, variance decomposition, and iterative analyses were performed using the “modEvA” and “vegan” packages to calculate the effects of each variable and area on species richness, respectively, after which a larger island was added to the analyses and a new dataset was built and repeated the construction of the quadratic equation and calculation of the degree of influence until the largest island was added to the dataset. We used generalized linear regression (GLM, family = Poisson) to analyze trends in the effects of each environmental variable on species richness during the iteration process, which were eventually visualized using the “ggplot2” package. In this process, the drivers of the SIE are the various environmental variables that have a decreasing effect on species richness [37]. The aforementioned analysis process was conducted in R version 4.2.3.

A total of three key hypotheses were chosen to explain the variables in this study: habitat diversity, nutrient replenishment, and disturbance. *PAR* and *LSI* represent multiple hypotheses in this study. Specifically, under equal area, patches with longer edges and more complex shapes create a greater diversity of habitats, increasing the opportunity for material exchange with the surrounding environment. However, these patches are also more susceptible to disturbance due to their greater exposure. Variables such as *Elevation* and the *Sealed_patch_* represent the habitat diversity hypothesis, reflecting the influence of physical and spatial patch characteristics on species diversity [14]. The nutrient replenishment hypothesis is also represented by the *DIS*, highlighting the potential for nutrient flow and species dispersal from the biodiversity pool [38]. Finally, the disturbance hypothesis includes variables related to anthropogenic influences, such as *Sealed*_50_, *AT*, *Light*, and *GDP*, which measure the degree of human impact on the remnant vegetation [39]. These variables provide a comprehensive framework for assessing the ecological drivers affecting species diversity and the small island effect (SIE) in urban remnant vegetation patches.

## 4. Discussion

Rapid urbanization in Qingdao has led to habitat fragmentation of remnant vegetation patches. The coastal and mountainous character of Qingdao has led to a special diversity pattern of remnant vegetation. In our study, we examined 17 remnant vegetation patches within the urban area, which only account for 0.32% of the total city area. Despite their small size, these patches support an impressive diversity, with 331 species recorded. Among these species, Asteraceae, Rosaceae, and Gramineae are the dominant families, and Asteraceae and Gramineae contain more genera and species. The most frequently observed genera included *Artemisia*, *Erigeron*, and *Rosa*. The most frequent species were *Trigonotis peduncularis*, *Oxalis corniculata*, *Robinia pseudoacacia*, and *Cocculus orbiculatus*. These results reflect that these species, especially those in the Asteraceae and Gramineae families, are more resilient and have a higher reproductive capacity, and can maintain high biodiversity even in fragmented urban green spaces, consistent with the results of studies in Yunnan [38], Harbin [40], Beijing [41], Shanghai [42]. Additionally, Asteraceae and Gramineae are usually introduced into cities due to their ornamental, economic, and medicinal values, which may also contribute to their spread and colonization [43,44]. These findings emphasize the importance of conserving remnant patches, which serve as valuable reservoirs of biodiversity despite their limited spatial footprint.

MacArthur and Wilson (1967) [15] hypothesized that the Small Island Effect (SIE) occurs when extinction events outnumber colonization events on small islands, resulting in a steeper decline in species richness as island size decreases. Our research in Qingdao’s urban remnant patches has provided empirical evidence to support this hypothesis, particularly for woody plants such as shrubs and trees. The variability in the expression of the SIE among different life forms of plants is a critical finding. While woody plants exhibit a clear response to the SIE, herbaceous plants do not, likely due to their superior dispersal capabilities and smaller size, which allow them to maintain higher population densities and lower extinction probabilities across patch sizes [45]. This distinction has significant implications for conservation strategies, suggesting that a one-size-fits-all approach may not be effective and that the specific ecological traits of plant communities must be considered.

The area thresholds of SIE identified for tree and shrubs—6.38 ha and 11.91 ha, respectively—underscore the necessity for larger remnant patches to sustain woody plant diversity. Additionally, smaller patches are more prone to SIE and experience a more rapid decline in diversity, thus rendering urgent and vital the rescue conservation efforts targeted at these threatened small patches that fall below these thresholds. Interestingly, total plant species does not exhibit an SIE until a much larger threshold of 86.44 ha is reached, which may be attributed to herbaceous plants mitigating the impacts of the SIE. Their presence in smaller patches compensates for the decline in woody plant diversity, helping to maintain higher overall species richness despite isolation effects. Schrader (2020) noted that species groups with high dispersal abilities or low extinction probabilities tend to show smaller breakpoint areas, as extinction rates only surpass colonization rates on very small islands [14]. To mitigate the effects of the SIE, remnant vegetation patches greater than 88.44 ha should be given priority for protection in the future urban planning of Qingdao, as they maintain a large amount of biodiversity, especially of woody plants, which are more vulnerable to the effects of habitat fragmentation. Therefore, when remnant patches are degraded to an area of 11.91 ha, urban planners and managers should prioritize allocating sufficient space to protect these remnant vegetation patches.

Environmental factors have been shown to play a significant role in influencing plant colonization and species richness, with several ecological theories proposed to explain the mechanisms behind the SIE. The habitat diversity hypothesis, for instance, suggests that the loss of certain habitat types below a critical area threshold can lead to the failure of dependent plant species to survive, which is supported by our findings for shrubs and trees [24]. As elevation increases in smaller patches, the terrain tends to become more uniform, limiting the diversity of microhabitats that are crucial for different species [46]. Additionally, smaller patches at higher elevations are more exposed to harsh climatic conditions like wind and temperature extremes, which can negatively impact plant survival and further reduce habitat diversity [15]. As the extent of sealed surfaces increases in a patch, critical habitat elements such as soil moisture, root space, and nutrient cycling are disrupted [47]. This limits the capacity of the patch to support diverse plant species, particularly those with specific habitat requirements like shrubs and trees. We note the result that species diversity increases with patch size when the *Sealed_patch_* variable increases. This result aligns with the habitat diversity hypothesis. As the impermeability (*Sealed_patch_*) within a patch increase, it is often accompanied by an increase in habitat types and habitat amounts, which in turn can lead to an increase in the number of species adapted to different habitat types. The surrounding sealed environments may also exacerbate the urban heat island effect [48], which can further degrade the internal conditions of the patch and make it less hospitable for certain plants. Furthermore, a species may exhibit positive, negative, and neutral responses depending on the type of edge encountered [49]. As the *PAR* and *LSI* amplifies the edge-to-interior ratio in smaller patches, leading to more pronounced edge effects and habitat degradation. These effects, such as increased light exposure, temperature variation, and wind exposure, can disrupt stable core habitat conditions that many woody plants depend on. The habitat amount hypothesis suggests that as *PAR* and *LSI* increase, so does the diversity of microhabitat types at the margins. This, in turn, leads to an increase in overall species diversity [50]. The greater edge effects introduced by a higher *PAR* and *LSI* create a more fragmented habitat, reducing core areas that provide essential resources like shelter from environmental stressors and stable conditions for growth [38]. These factors ultimately limit specific habitats that are indispensable for certain plant species, leading to a decline in species that require more stable or specialized habitats, while widespread species capable of thriving in both edge and central areas are less impacted by these changes.

The nutrient replenishment hypothesis posits that exogenous resources in the vicinity of an island can increase island productivity, thereby affecting the number of species on the island [51]. In both shrubs and trees, *PAR*, *LSI*, and *DIS* showed clear trends; the greater the edge effect from *PAR* and *LSI*, the greater the potential for exchanging material with the outside world and the more nutrients received [52]. Under a certain area threshold, the patch itself is small in size and more likely to receive nutrients, so the edge effect has little effect on nutrient supplementation, but as the patch area increases, the nutrient supplementation becomes insignificant, and at this time, the edge effect plays a positive role and is able to provide nutrient supplementation for the larger island, which plays a positive role in plant survival [27]. Our species composition in remnant patches (in our case, remnant patches could serve as habitat islands) showed a high degree of overlap with the plant species observed in Mt. Laoshan (in this case, Mt. Laoshan, which serves as the species pool), This result aligns with the classic theory of island biogeography theory, which posits that when habitat islands are closer to “mainland”, they have a higher possibility to obtain nutrient replenishment and species dispersal from the species pool [15]. Consequently, enhancing the ecological quality of Mt. Laoshan is likely to have a beneficial impact on the protection of plant species diversity in the remnant vegetation patches within the main urban area.

The disturbance hypothesis, which aligns with our findings, posits that small islands, analogous to fragmented habitat patches, may be in various stages of recovery from disturbance, with transient or random events potentially reducing the expected positive relationship between area and species richness [53]. Our findings indicate that in smaller patches, edge effects associated with *PAR* and *LSI* intensify disturbance, leading to a decline in species numbers [54]. This is particularly relevant as it suggests that the configuration of habitat patches can significantly influence biodiversity. The *DIS*, *Sealed_50_*, *AT*, *Light*, and *GDP* indices, which serve as indicators of urbanization intensity, further highlight the multifaceted nature of disturbance, with higher values correlating with increased disturbance around remnant patches [55]. Small patches are more susceptible to external disturbance and can also exhibit higher biodiversity, which can act as a conduit for species movement and gene flow. However, this comes at the cost of increased vulnerability to disturbance that can rapidly alter habitat conditions, thus affecting species survival and overall biodiversity [56]. These insights are crucial for biodiversity conservation efforts, especially in the context of urbanization where habitat fragmentation is a significant concern.

In summary, the SIE is a complex phenomenon influenced by a multitude of factors, including plant traits and environmental conditions. In order to better conserve biodiversity, we should maintain habitat diversity and avoid anthropogenic disturbance as much as possible, which can be achieved by means of increasing the area of green space and reducing the destruction of pristine habitats. At the same time, Mt. Laoshan and the forests around the remnant vegetation patches are important to maintain the species diversity of the remnant vegetation patches, so attention should be paid to the protection of the green space environment around the patches. The results of our study are of great significance for the conservation of biodiversity and provide a scientific reference value for the conservation strategy for different life forms of vegetation. Given the limited number of remnant natural patches within Qingdao’s urban areas, our study focused on a sample of 17 patches, which provides a fundamental perspective on the Small Island Effect (SIE) in this region. Future research endeavors should expand this analysis to encompass a broader range of urban environments and allow for a more comprehensive understanding of the drivers behind SIE. Additionally, investigating the differences in the SIE between natural remnants and artificially created green spaces could offer further insights into the mechanisms of the SIE and its implications for urban ecology and conservation efforts. This expanded scope of research can also explore how the configuration and connectivity of habitat patches influence biodiversity and ecological processes within urban landscapes. By doing so, we can enhance our predictive capacity regarding the impacts of urbanization on native species and work towards more effective biodiversity conservation strategies in the face of habitat fragmentation.

## 5. Conclusions

This study detected the Small Island Effect (SIE) in Qingdao’s urban remnant vegetation, particularly in woody plants, with significant area thresholds identified for trees (6.38 ha) and shrubs (11.91 ha). Then, trends in the contribution of the environmental variables to changes in species richness over the iterations were analyzed. Our findings revealed the following: (1) In total, 331 species were recorded in 17 remnant vegetation patches, proving that remnant vegetation patches can maintain a high number of remnant vegetation species in urban areas. (2) The SIE was only detected for woody plants, with a threshold area of 6.38 ha for trees and 11.91 ha for shrubs, suggesting woody plants are more sensitivite than herbaceous plants to the SIE. (3) The drivers of the SIE differ between shrubs and trees. The species diversity of total plants is largely influenced by *Sealed*_50_, *DIS*, *GDP*, *LSI*, and *AT;* shrub diversity is more sensitive to *LSI*, *Sealed_patch_*, and *PAR;* changes in tree diversity are more strongly correlated with *DIS*, *GDP*, *Light*, and *PAR*. This study supports the habitat diversity, disturbance, and nutrient replenishment hypotheses, emphasizing the need for targeted conservation strategies that consider the specific ecological traits of plant communities. In light of the aforementioned findings, it is recommended that the conservation of different life forms is differentiated in urban planning and development to enhance the maintenance of native habitats of remnant vegetation patches.

Remnant vegetation is suitable for creating near-natural landscapes because of its high spontaneity, low maintenance costs, and ornamental values. The use of remnant vegetation in the design of urban artificial green spaces will yield higher ecological and economic benefits. The attributes of the patches, such as patch area, patch perimeter, landscape shape index, and perimeter–area ratio, which are related the size of the habitat island, edge effects, and habitat quality, have a significant influence on the species diversity of the remnant vegetation. When constructing and managing urban green spaces, urban planners should consider these properties to enhance the species richness of remnant vegetation and enrich the urban green spaces, especially for patches located away from natural species pool. The green patches should have the smallest possible perimeter-to-area ratio; large patches should be protected because they have greater species conservation capacity. However, due to the severe shortage of urban land, it is impractical to design large patches when planning green spaces in urban areas; but it seems feasible and effective to utilize more small patches to construct ecological corridors to promote vegetation dispersal. In addition, external disturbance is highly disruptive to plant species richness, which means that urban planners and managers may need to take additional specific measures to reduce this risk, e.g., by reducing anthropogenic disturbance such as cutting, trampling, building, and cultivation. Our insight into patterns of remnant vegetation richness in Qingdao is of great significance for biodiversity conservation and provides scientific reference value for the conservation strategies for different life forms of vegetation and urban landscape design.

## Figures and Tables

**Table 1:** Species–area relationship fitting results for 17 remnant vegetation patches vegetation. AICc, corrected Akaike information criterion; C, intercept; K, number of estimable parameters; T, area threshold; Z_1_, first slope; Z_2_, second slope. The number of models; please check Table 3.

Group	Models	Parameters				Model Comparison		
		C	Z_1_	Z_2_	T	K	AICc	ΔAICc
Total plants	5	−138.068	40.408	0	0	2	149.04	0
	1	−25.023	16.308	31.382	4.926	3	150.654	1.613
	2	−179.611	16.308	47.689	4.926	3	150.654	1.613
	3	66.875	186.37	0	6.216	2	150.998	1.958
	4	−1091.611	186.37	0	6.216	2	150.998	1.958
	6	72.941	0	0	0	1	166.259	17.218
Annual herb	5	−24.248	8.586	NA	NA	2	103.611	0
	1	−24.328	8.603	−0.026	5.126	3	106.6	2.989
	2	−24.195	8.603	8.577	5.126	3	106.6	2.989
	3	19.5	33.434	NA	6.216	2	110.172	6.561
	4	−188.325	33.434	NA	6.216	2	110.172	6.561
	6	20.588	NA	NA	NA	1	116.198	12.587
Perennial herb	5	−56.323	14.717	NA	NA	2	112.715	0
	1	23.465	−3.211	18.839	4.506	3	114.95	2.235
	2	−61.426	−3.211	15.628	4.506	3	114.95	2.235
	3	18.375	66.19	NA	6.216	2	116.54	3.825
	4	−393.063	66.19	NA	6.216	2	116.54	3.825
	6	20.529	NA	NA	NA	1	131.324	18.609
Shrub	3	6.804	10.188	—	4.216	2.000	115.033	0.000
	4	−36.147	10.188	—	4.216	2.000	115.033	0.000
	5	−35.959	10.153	—	—	2.000	115.098	0.065
	1	−9.808	4.794	10.478	5.346	3.000	115.852	0.819
	2	−65.823	4.794	15.272	5.346	3.000	115.852	0.819
	6	17.059	—	—	—	1.000	124.510	9.476
Tree	3	7.742	6.977	—	4.216	2.000	107.947	0.000
	4	−21.674	6.977	—	4.216	2.000	107.947	0.000
	5	−21.539	6.952	—	—	2.000	107.999	0.051
	1	7.882	0.754	8.868	5.046	3.000	109.413	1.465
	2	−36.867	0.754	9.622	5.046	3.000	109.413	1.465
	6	14.765	—	—	—	1.000	114.729	6.782

**Table 2:** The description of explanatory variables in the GLM.

Explanatory Variables	Explanatory Variables	Source	Proxy Hypothesis
*Elevation*	Elevation	Resource and Environmental Science Data Platform (resolution = 30 m, https://www.resdc.cn (accessed on 1 October 2023))	Habitat diversity
*PAR*	Perimeter–area ratio	$PAR=\frac{Patch\;Perimeter}{Patch\;Area}$	Nutrient replenishment, Habitat diversity, Disturbance hypothesis
*LSI*	Landscape shape index	$LSI=P/2\sqrt{\pi A}$	Nutrient replenishment, Habitat diversity, Disturbance hypothesis
*DIS*	Distance to the source of species	Resource and Environmental Science Data Platform (resolution = 30 m, https://www.resdc.cn (accessed on 1 October 2023))	Nutrient replenishment. Disturbance hypothesis
*Sealed_patch_*	The proportion of sealed surface within the patch	National Geomatics Center of China (Resolution = 30 m, https://www.webmap.cn/ (accessed on 1 October 2023))	Habitat diversity
*Sealed*_50_	The proportion of sealed surface around the patch within a radius of 50 m	National Geomatics Center of China (Resolution = 30 m, https://www.webmap.cn/ (accessed on 1 October 2023))	Disturbance hypothesis
*AT*	Anthropogenic disturbance, class 1 when there is less than one type of interference and the intensity of the interference is small, class 2 when there are one or two types of low-level interference, class 3 when there are three types of medium-level interference, and class 4 when there are four or more types of high-level interference.	(Hong Liang, 2022 [34])	Disturbance hypothesis
*Light*	The annual average night light intensity of China in 2020	Geographic remote sensing ecological network platform (Resolution = 30 m, www.gisrs.cn (accessed on 1 October 2023))	Disturbance hypothesis
*GDP*	The Gross Domestic Product data of China in 2020	Geographic remote sensing ecological network platform (Resolution = 30 m, www.gisrs.cn (accessed on 1 October 2023))	Disturbance hypothesis

**Table 3:** Detection model for small island effects.

Model	Equation	Description
(1)	S = C + (log A ≤ T) × Z_1_ × log A + (log A > T) × ((Z_1_ × T + Z_2_ × (log A − T))	Regression fragments and slope iteration fragments
(2)	S = C + (log A ≤ T) (Z_1_ × log A + (Z_2_ − Z_1_) × T) + (log A > T) × Z_2_ × log A	Regression fragments and slope iteration fragments
(3)	S = C + (log A > T) × Z_1_ × (log A − T)	Zero-slope regression fragments and slope iteration fragments
(4)	S = C + (log A ≤ T) × Z_1_ × T + (log A > T) × Z_1_ × log A	Regression fragment and direct inheritance fragment
(5)	S = C + Z_1_ × log A	Linear regression
(6)	S = C	Zero-slope regression

Note: In these equations, S stands for species richness and A for area, while c (intercept), zi (slope), and T (break point) are fitted parameters. Z_1_ and Z_2_ denote slopes of first and second non-horizontal segmentation regression lines. In this study, the minimum residual sum-of-squares method was employed for the estimation of the thresholds, and the iterative method was utilized for the nonlinear estimation, with an increase of 0.001 for each iteration. The threshold T corresponding to the minimum residual sum-of-squares was selected as the minimum area threshold.

## Data Availability

The data presented in this study are openly available in FigShare at https://doi.org/10.6084/m9.figshare.27620280.v1 (Posted on 6 November 2024).
